# Analysis of prognostic factors in patients with brain metastases affecting survival

**DOI:** 10.1186/s43046-022-00146-z

**Published:** 2022-11-01

**Authors:** Sweety Gupta, Sumit Singh, Atokali Chophy, Sharanya Nair, Rachit Ahuja, K. Kusum, Deepa Joseph, Rajnish Arora, Amit Gupta, Manoj Gupta

**Affiliations:** 1 Department of Radiation Oncology, AIIMS Rishikesh, Rishikesh, India; 2 College of Nursing, AIIMS Rishikesh, Rishikesh, India; 3 Department of Neurosurgery, AIIMS Rishikesh, Rishikesh, India; 4 Department of Surgery, AIIMS Rishikesh, Rishikesh, India

**Keywords:** Metastases, Brain, Whole brain radiotherapy, Survival

## Abstract

**Introduction:**

Brain metastases (BM) are associated with dismal prognosis as they cause significant morbidity and affect the quality of life of patients. Management of BM depends on the following factors: age, patient performance, size and the number of lesions, location of the tumor, comorbidities, primary tumor type, and extracranial disease burden. In the present study, the pattern of occurrence, clinical characteristics, treatment outcome of brain metastases, and factors, tumor characteristics, and treatment that may impact BM patients’ overall survival were analyzed.

**Methods:**

Retrospective analysis of medical records of 116 patients with histologically proven primary site solid tumors with brain metastases was done in the present study. Clinicoradiological and pathological parameters were documented. The relationship between variables and outcome was assessed by univariate analysis using the Cox proportional regression model to reach a significance of *p* < 0.05, to determine independent predictors of overall survival.

**Results:**

One hundred sixteen patients of BM from various solid malignancies were included. Age ranged from 18 to 81 years (median 53.5). One hundred four patients received WBRT with a dose range of 8–40Gy/1–15fr, 7 received SRS with a dose of 18–24Gy depending on the size of the metastatic lesion, and 2 received SRT 27–33Gy/3fr. At the time of final analysis, 47 patients with BM had expired, 60 were lost to follow-up, and 9 were alive. Median survival was 8.25 (0.5–32.5 months) months. Female gender (*χ*^2^ = 8.423; *p* = 0.015), RPA I (*χ*^2^ = 9.353; *p* = 0.05), and metachronous BM (*χ*^2^ = 3.793; *p* = 0.03) were associated with better survival. Patients with age 41–50 years, adenocarcinoma lung histology, and supratentorial location survived more than 2 years but did not show any statistical significance.

**Conclusion:**

Brain metastases portend a very dismal prognosis. Certain clinicoradiological and pathologic factors have been identified to affect survival. More prospective multicentric trials, with a larger sample size, need to be conducted to assess the benefit of radiation in patients with limited life expectancy and identify prognostic and predictive factors for survival.

## Introduction

Brain metastases (BM) are the most common intracranial malignancy and ten times more common than primary brain tumors [1]. Lung primary is most commonly associated with the occurrence of brain metastases (40–50%), followed by breast (15–30%), melanoma (5–20%), colorectal cancer (CRC) (3–8%), and renal cell cancer (2–4%) [2]. Small cell lung cancer and adenocarcinoma are the common histologies in lung cancer associated with BM. Brain metastases are associated with dismal prognosis as they cause significant morbidity and affect the quality of life of patients [3]. Management of BM depends on the following factors: age, patient performance, size and the number of lesions, location of tumor, comorbidities, primary tumor type, and extracranial disease burden. Commonly used treatment modalities include surgery, whole brain radiotherapy (WBRT), stereotactic surgery (SRS) and radiotherapy, chemotherapy, and targeted therapies [4]. Quality of life of patients and the long-term toxicity and management complications should also be carefully balanced when deciding on the treatment of BM patients. WBRT is associated with an increment in intracranial tumor control rates but with long-term neurocognitive decrement, and without improvement in overall survival, SRS is usually considered in patients with a limited number of BM [5, 6].

The literature is sparse on the factors that may affect survival in patients with brain metastases. We conducted a single-center retrospective study to assess the pattern of occurrence, clinical characteristics, and management outcome of brain metastases and also identified clinical factors, tumor characteristics, and treatment that may impact BM patients’ overall survival.

## Methods

### Patient selection

We retrospectively analyzed medical records of a total of 116 patients of solid tumors with brain metastases who received treatment between Jan 2018 and March 2021. Patients with a histologically proven primary site (solid tumors) with brain metastases were included. Patients who had leptomeningeal metastases and hematological malignancies were excluded. Approval was taken from the Institutional Ethics committee. Diagnosis of brain metastases was made on the basis of the following criteria: imaging evidence of intracranial metastases or pathological confirmation of a metastatic brain tumor.

### Study variables

Parameters documented were age, gender, primary site, stage, primary tumor type, date of diagnosis of brain metastases, synchronous/metachronous, and clinical presentation of BM; the number of lesions; location, number of extracranial metastases, RPA-I:KPS ≥70%, age <65 years, controlled primary site, and no extracranial metastases; RPA-II: all others; RPA-III:KPS < 70; surgery for metastases: yes/no; radiotherapy technique, dose, time after brain metastases (in months); and status of the patient at the time of analysis.

### Outcome

The primary outcome assessed was overall survival defined as the time interval between BM diagnosis and death from any cause. The secondary outcome assessed was clinical factors, tumor characteristics, and treatment that may impact the overall survival of BM patients.

### Statistical analysis

The method of analysis of all subjects was intention-to-treat analysis. Relationships between categorical variables were analyzed using Fisher’s exact test, while continuous data was analyzed using the Mann–Whitney *U* test. The relationship between variables and outcome was assessed by univariate analysis using the Cox proportional regression model to reach a significance of *p* < 0.05, to determine independent predictors of overall survival. Overall survival and median survival were calculated. Patients who were lost to follow-up were excluded from the survival analysis.

## Results

### Patient characteristics

In the present study, 116 patients of BM from various solid malignancies were included during the study period. Age ranged from 18 to 81 years (median 53.5). The most common age group was 41–50 years with 38 patients. Male to female ratio was 1.57. Lung cancer was the most common primary malignancy 80 (68.9%) with 35% being adenocarcinoma histology followed by breast cancer 23 (19.8%) patients. Thirty-one (26.7%) patients had bone metastases. The most common presenting symptom was headache in 64 patients, and 7 had an incidental diagnosis of brain metastases, in whom brain MRI was done as part of staging workup for advanced malignancy. Out of seven patients with asymptomatic BM, six were of lung cancer (SCLC and NSCLC) and one breast cancer. According to RPA classification, 51, 15, and 50 patients were of I, II, and III respectively. Multiple lobes of the brain were involved in 51.7% of patients. The time to diagnosis of BM was more than 6 months (metachronous) from primary malignancy in 73 patients whereas 43 patients had synchronous BM. The median follow-up time was 6 months (Table 1).

**Table 1:** Clinical characteristics of patients with BM (*n* = 116)

Variables	Options	Number (%)
Age	18–30 years	04 (3.4)
	31–40 years	07 (6.0)
	41–50 years	38 (32.8)
	51–60 years	35 (30.2)
	61–70 years	25 (21.6)
	More than 70 years	07 (6.0)
Gender	Male	71 (61.2)
	Female	45 (38.8)
Primary site of cancer	Lung	80 (68.9)
	Breast	23 (19.8)
	Gynecological	03 (2.58)
	Soft tissue sarcoma	02 (1.72)
	Gastrointestinal	02 (1.72)
	Genitourinary	03 (2.58)
	Others	03 (2.58)
Histopathology	SCLC	20 (17.2)
	Adenocarcinoma	41 (35.3)
	Squamous	21 (18.2)
	Others	34 (29.3)
Extracranial metastasis	Liver	09 (7.8)
	Bone	3 (26.7)
	Adrenal	05 (4.3)
Clinical presentation of brain metastasis	Headache	64 (55.3)
	Seizures	15 (12.9)
	Weakness	30 (25.8)
	Asymptomatic	07 (6.0)
RPA	I	51 (43.9)
	II	15 (13.0)
	III	50 (43.1)
Number of BM	Single	30 (25.8)
	Multiple	86 (74.2)
Location of BM	Cerebrum	50 (43.1)
	Cerebellum	06 (5.2)
	Multiple	60 (51.7)
Time of occurrence of BM	Synchronous	43 (37.1)
	Metachronous	73 (62.9)

### Treatment and outcome

Patients with synchronous BM were treated with radiation therapy followed by systemic chemotherapy depending on the performance status of the patient. None of the patients had undergone surgery prior to radiation. One hundred four patients received WBRT with a dose range of 8–40Gy/1–15fr, 7 received SRS with a dose of 18–24Gy depending on the size of the metastatic lesion, and 2 received SRT 27–33Gy/3fr. At the time of the final analysis, 47 patients with BM had expired, 60 were lost to follow-up, and 9 were alive (Table 2).

**Table 2:** Treatment and outcome of BM patients

Variables	Options	Number (%)
Radiation therapy	WBRT	104 (89.6)
	WBRT + Boost	03 (2.6)
	SRS	07 (6.0)
	SRT	02 (1.8)
Dose of radiation therapy	18–24Gy/1 fr	07 (6.0)
	27–33Gy/3fr	02 (1.8)
	8–40Gy/1–15 fr	107 (92.2)
Present status	Alive	09 (7.8)
	Death	47 (40.5)
	Lost to follow-up	60 (51.7)
OS (after BM)	Less than 6 months	22 (18.9)
	6–12 months	18 (15.5)
	1–2 years	07 (6.0)
	More than 2 years	09 (7.8)

**Fig. 1:**
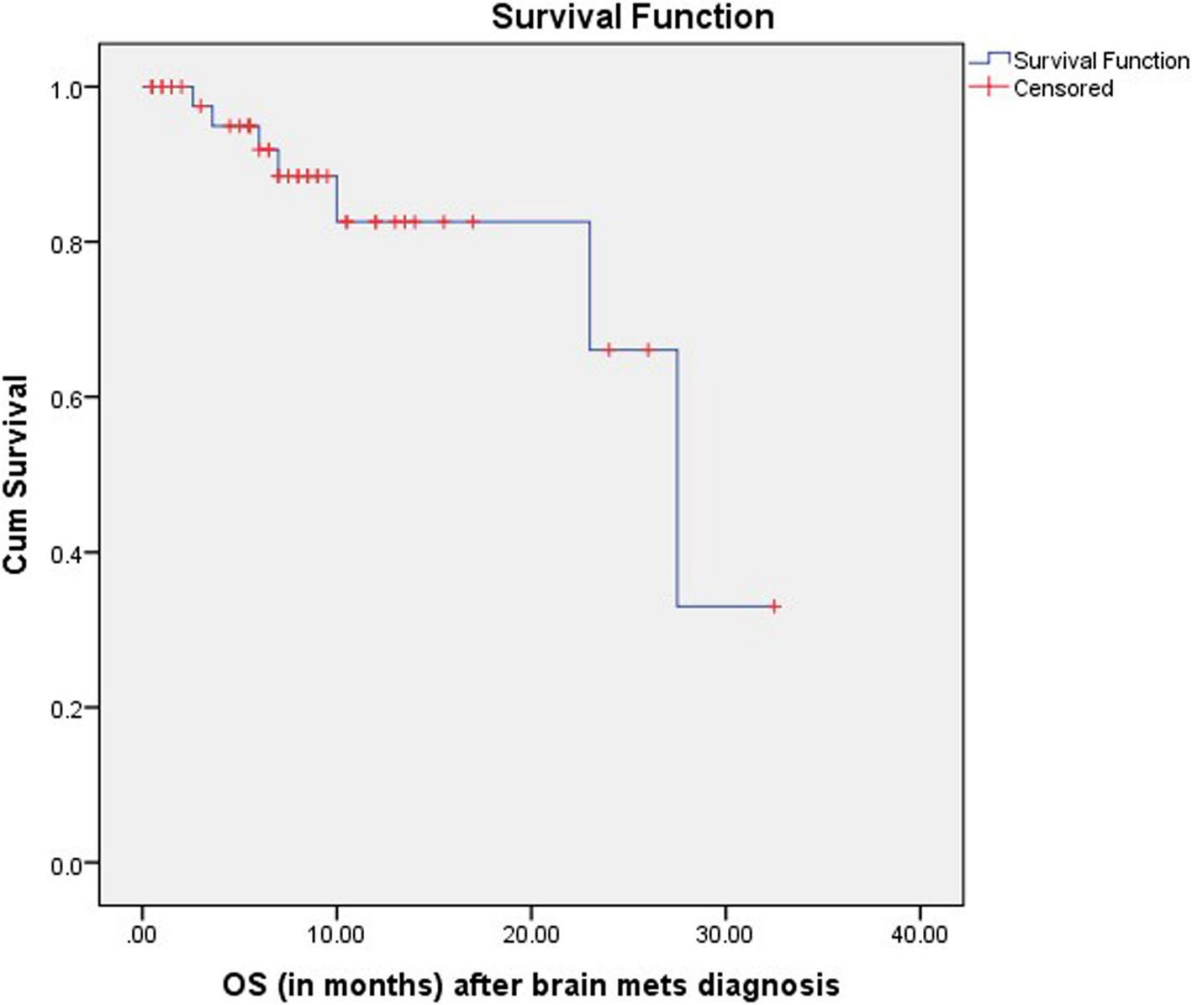
Kaplan–Meier survival plot of BM patients

A large number of patients being lost to follow-up might be due to the COVID-19 situation prevailing for the last 2 years. The cause of death included disease progression and aspiration pneumonia. Twenty-two patients survived for less than 6 months whereas 16 for more than 1 year. Median survival was 8.25 (0.5–32.5 months) months (Fig. 1).

**Fig. 2:**
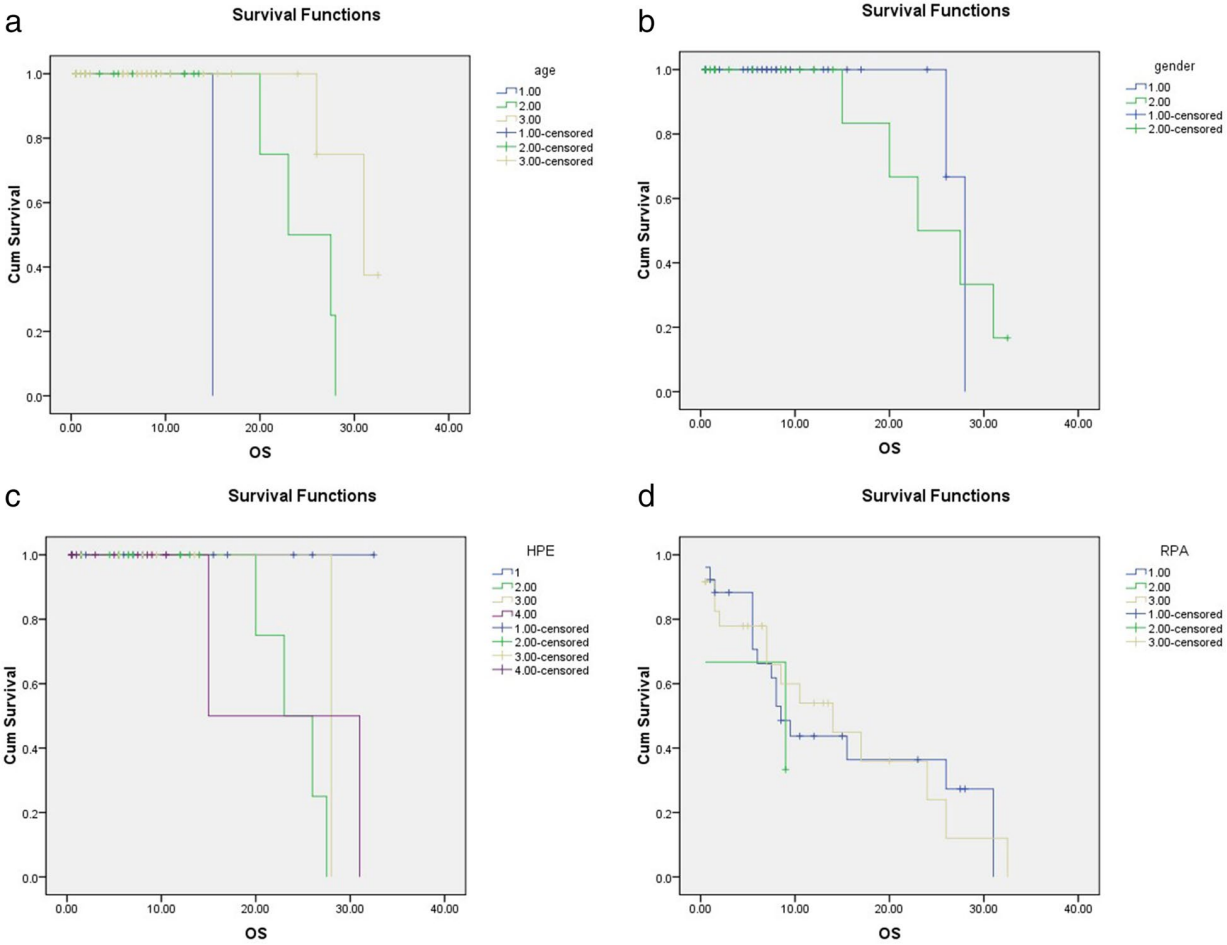
**a**–**d** Survival analysis (in months) by the Kaplan–Meier method for variables **a** age, **b** gender, **c** histology, and **d** RPA class

Overall median survival was high in age more than 50 years (32 months), in male (28 months) adenocarcinoma histology in lung cancer (32 months), and in KPS scores more than 70 (32 months) (Fig. 2a–d).

### Factors affecting survival

Correlation of variables was done with survival data, and female gender (*χ*^2^ = 8.423; *p* = 0.015), RPA I (*χ*^2^ = 9.353; *p* = 0.05), and metachronous BM (*χ*^2^ = 3.793; *p* = 0.03) were associated with better survival. Patients with age 41–50 years (4), adenocarcinoma histology (4), multiple brain metastases (7), and supratentorial location (4) survived more than 2 years and did not show any statistical significance (Table 3).

**Table 3:** Correlation of factors with survival in BM patients

Variable	Present status				Chi-square	***p***-value
		Alive	Dead	Lost to follow-up		
Age (in years)	18–30	1	0	3	7.747	.654
	31–40	0	2	5		
	41–50	4	15	19		
	51–60	2	17	16		
	61–70	2	11	12		
	>70	0	2	5		
Gender	Male	2	34	35	8.423	.015*
	Female	7	13	25		
Histopathology	SCLC	0	12	8	5.945	.429
	Adeno	4	14	23		
	Squamous	1	9	11		
	Others	4	12	18		
Extracranial metastases	Liver	1	3	5	2.409	.966
	Bone	2	11	18		
	Adrenal	1	2	2		
	Multiple	3	22	24		
	None	2	9	11		
Clinical presentation of brain Mets	Headache	4	26	34	5.618	.467
	Seizure	1	3	11		
	Weakness	3	14	13		
	Asymptomatic	1	4	2		
RPA	I	7	20	24	9.353	.05*
	II	0	3	12		
	III	2	24	24		
Number	Single	2	13	15	0.165	.921
	Multiple	7	34	45		
Location	Cerebrum	4	23	23	3.544	.738
	Cerebellum	0	2	4		
	Multiple	5	22	31		
Synchronous/metachronous	Synchronous	1	16	26	3.793	.03*
	Metachronous	8	31	34		
RT	WBRT	8	41	55	2.716	.844
	WBRT+ Boost	0	2	1		
	SRS	1	3	4		
	SRT	0	1	0		

## Discussion

Brain metastases are the most common intracranial neoplasms and occur in 15–40% of patients with systemic cancer [7]. Incidence is on the rise in the last 20 years because of the upsurge in primary malignancies, e.g., lung and breast cancer, and newer therapeutic modalities increasing survival and imaging modalities which can identify brain metastases [8]. The more common age of BM occurrence is the 5th to 7th decade. Similarly, in the present study, the median age was 53 years with 57.7% of patients being more than 50 years of age. BM are common in the lung (36–64%), breast (15–25%), and melanoma (5–20%) followed by less common colorectum, genitourinary malignancies [9, 10]. In the present study also, lung cancer was the most common primary malignancy in 68.9% of patients with 35% being adenocarcinoma histology followed by breast cancer in 19.8% of patients. BM are primarily diagnosed as metachronous, and less common synchronous (30%), and in few patients, BM may be the presenting symptom in occult cancer (10%). In the present study, 62.9% of patients had metachronous BM presentation [11].

The common symptoms of BM include headache, vomiting, seizures, and weakness which are due to mass effect, edema, and raised intracranial pressure. In this study, 55.3% of patients presented with headache followed by seizures and weakness whereas 6% did not have any symptoms pertaining to BM.

The decision regarding treatment is usually done on the basis of the recursive partition analysis (RPA) prognostic scale which was developed by Gaspar et al. in 1977 [12]. This is based on four prognostic factors: performance according to the Karnofsky Performance Scale (KPS), control of the primary disease, presence or absence of extracranial disease, and age greater or less than 65 years. Patients are divided into three classes I, II, and III with an estimated survival of 7.1, 4.2, and 2.3 months respectively. Patients with RPA I and II are considered for surgery or radiation of BM whereas class III is usually considered for supportive care. In this study, 43.9% of patients were of RPA class I and 43.1% were class III. Also, at the time of analysis, 7 patients in class I and 2 patients in class III were alive, suggesting other factors might also be associated with increased survival in some patients. Though the prognostic scale suggests survival, also, there is disease-specific graded prognostic assessment (DS-GPA) in which other prognostic factors specific to each malignancy are considered, e.g., molecular subtype in breast cancer and the number of metastases in the lung, renal carcinoma, and melanoma [13, 14].

Management strategies for BM include surgery, whole brain radiotherapy (WBRT), stereotactic surgery (SRS), and stereotactic radiotherapy (SRT) [15]. Dose for WBRT varies from 30Gy in 2 weeks to 20Gy in 1 week without any difference in symptom control and overall survival in the two fractionations [16]. 89.6% of patients in this study received WBRT, with the most common fractionation being 20Gy in 5 fractions because 74.2% of patients had multiple metastases. There was no immediate toxicity due to this dose fractionation but a longer neurological assessment is required to identify any effect on neurocognitive function. Only 6% and 1.8% of patients received SRS and SRT respectively.

Patients with good performance status, a limited number of brain lesions, and controlled extracranial primary should be considered for SRS and have shown 80–90% local control with symptom improvement and median survival of 6–12 months [17]. The median survival in the present study was 8.25 months. Ekici et al. in their study of BM had a median survival of 6.7 months. They also identified patients with KPS ≥ 70, with single BM, with extracranial primary controlled, and without leptomeningeal metastases [18].

Similarly, in the present study, patients with RPA I class survived more than II and III, but there was no correlation in patients with number of brain lesions, extracranial primary status, or disease burden elsewhere and survival. Lock et al. assessed whether patients with shorter expected survival would benefit from WBRT and concluded that poor performance status and the extent of metastatic burden could be a predictor of early death in patients with BM [19]. Similarly, in the present study, patients with synchronous BM, RPA-II, and male gender had poor survival, but there was no difference on OS depending on the WBRT dose. Rastogi et al. identified that female gender, performance status, breast primary, metachronous BM, solitary lesion, and controlled primary were associated with better survival in BM [20]. In the present study, results regarding factors affecting survival in BM also showed a similar trend as mentioned in previous studies but some did not show any statistical significance.

The limitations of the present study being disease-specific graded prognostic assessment for each disease site could have revealed more factors affecting survival. A large number of patients were lost to follow-up due to the COVID-19 situation at the time of analysis, which might have affected our results. But more prospective multicentric trials, with a larger sample size, need to be conducted to assess the benefit of radiation in patients with limited life expectancy and identify prognostic and predictive factors for survival.

## Conclusion

Brain metastases portend a very dismal prognosis. WBRT remains the cornerstone of the management of multiple brain metastases, whereas for solitary lesion, surgical excision followed by SRS or SRT is the preferred treatment modality. The performance status of the patient with extracranial disease burden plays an important role in decision-making. In the present study, female gender, RPA I class, and metachronous brain metastases were identified as factors affecting the overall survival of BM patients. Also, patients with age 41–50 years, adenocarcinoma histology, and supratentorial location of tumors survived longer though the results were not statistically significant.

## Data Availability

The data that support the findings of this study are available on request from the corresponding author.

## Abbreviations

BM: Brain metastases
CRC: Colorectal cancer
WBRT: Whole brain radiotherapy
RPA: Recursive partitioning analysis
SRS: Stereotactic surgery
SRT: Stereotactic radiotherapy
KPS: Karnofsky Performance Status
SCLC: Small cell lung cancer
NSCLC: Nonsmall cell lung cancer
DS-GPA: Disease-specific graded prognostic assessment DS-GPA
